# Supplementary material for: Tumour-Associated MUC1 Exerts Multiple Effects on Cholesterol and Lipid Metabolism—A Potential Pathogenic Effector of Atherosclerosis in Cancer
Source: Int J Mol Sci. 2026 Jan 4;27(1):518. doi: 10.3390/ijms27010518 (PMC12787243; doi:10.3390/ijms27010518)
Supplement: Supplementary file 1 [file ijms-27-00518-s001.zip › ijms-3933542-supplementary.pdf]

Supplementary File S1

The density ratio of Western blot results

The bar graphs show the expression and densitometric ratios of each protein relative to GAPDH, AKT or ERK. The mean ratios + S.E. from triplicate identical blots were calculated for each bar.

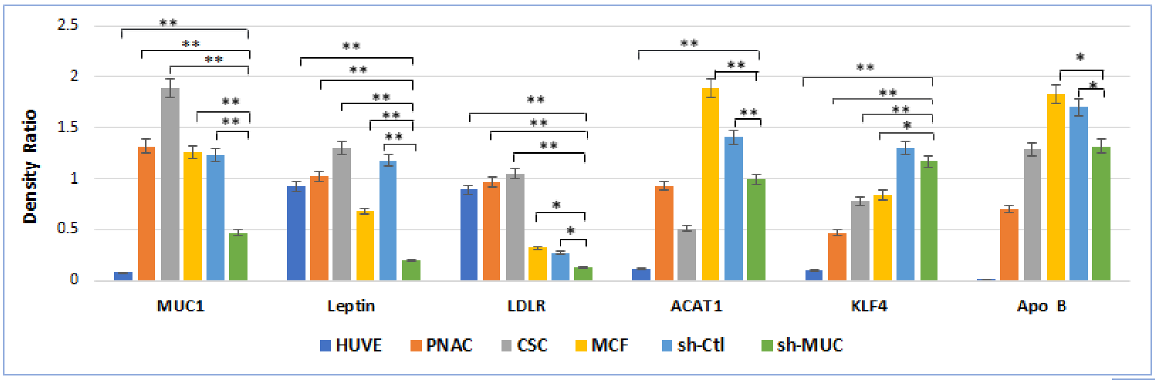

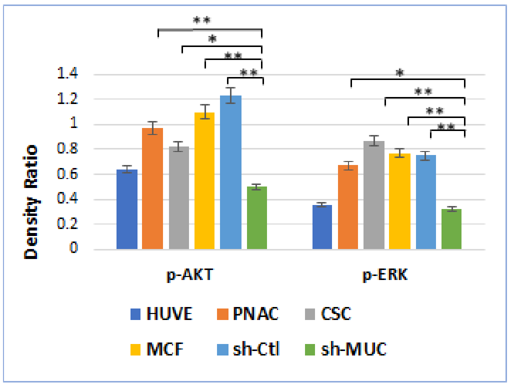

For figure 2A. Western blot assay. Signaling proteins and proteins involved in cholesterol metabolism and atherosclerosis pathogenesis correspond according to the TA-MUC1 level of cancer cells. The ratio of protein band density was calculated in respect of the level of GAPDH, AKT or ERK. t-test used to determine significance, * $P < 0.05$, ** $P < 0.01$ for sh-MUC1 cells respectively compared with other cells.

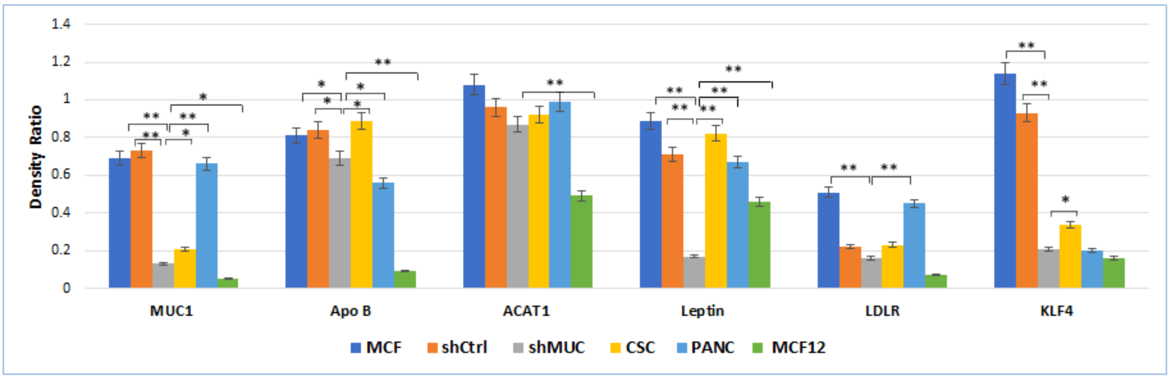

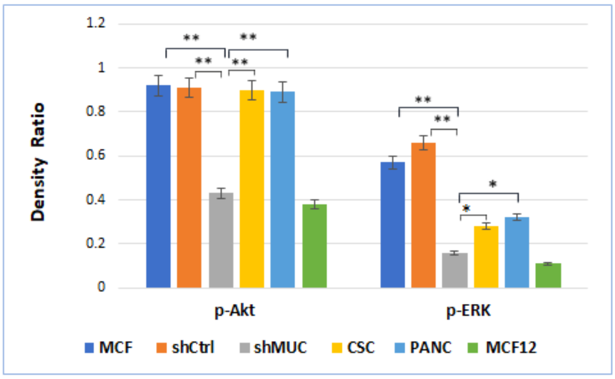

For figure 2B. The levels of signaling proteins and proteins involved in cholesterol metabolism and atherosclerosis pathogenesis were also liable to induction by sEVs from cancer cells when added to normal HUVEC. The density ratio of Western blot results was calculated in respect of the level of GAPDH, AKT or ERK. t-test used to determine significance, * $P < 0.05$, ** $P < 0.01$ for sh-MUC1 cells respectively compared with other cells.

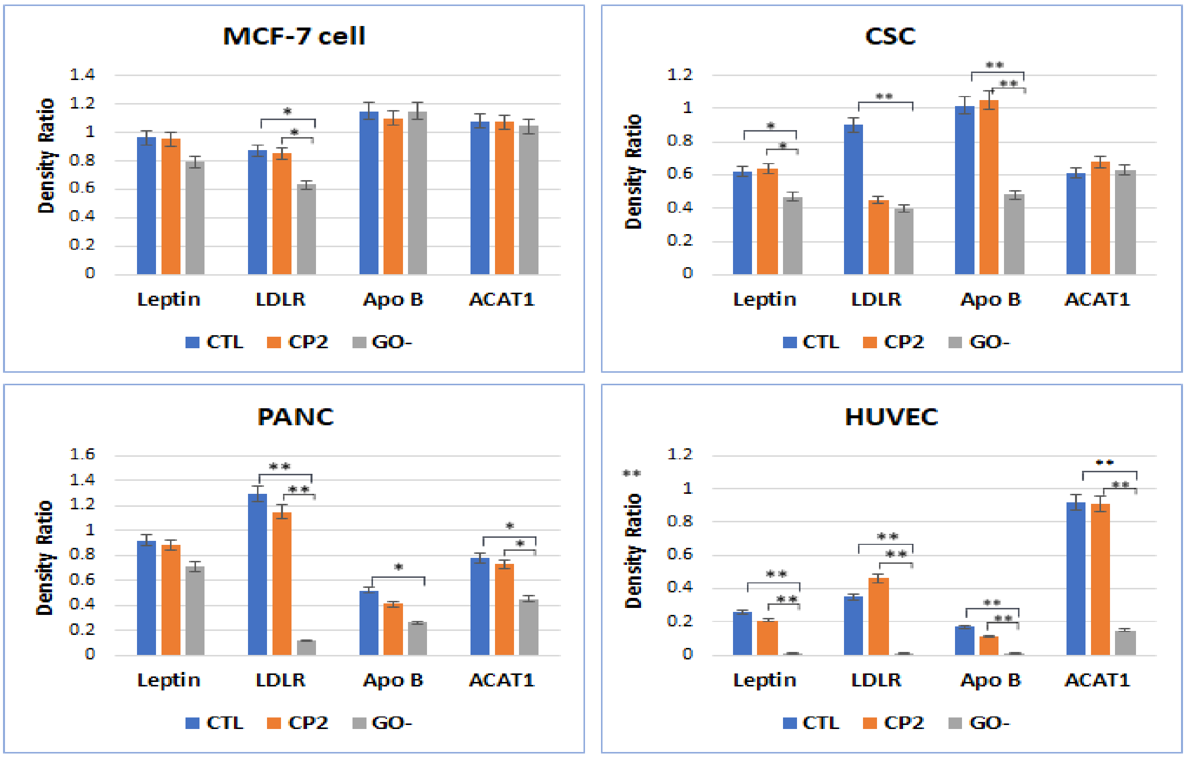

For figure 3A. Western blot of TA-MUC1 effect on cholesterol metabolism within different cancer cells is liable to restraint by the MUC1 inhibitor GO-203 compared with a control peptide (CP2) treated and untreated cells (CTL). The density ratio of Western blot band was calculated in respect of the level of GAPDH. t-test used to determine significance, * $P < 0.05$, ** $P < 0.01$ for inhibitor GO-203 treated cells respectively compared with CTL and control peptide CP2 treated cells.

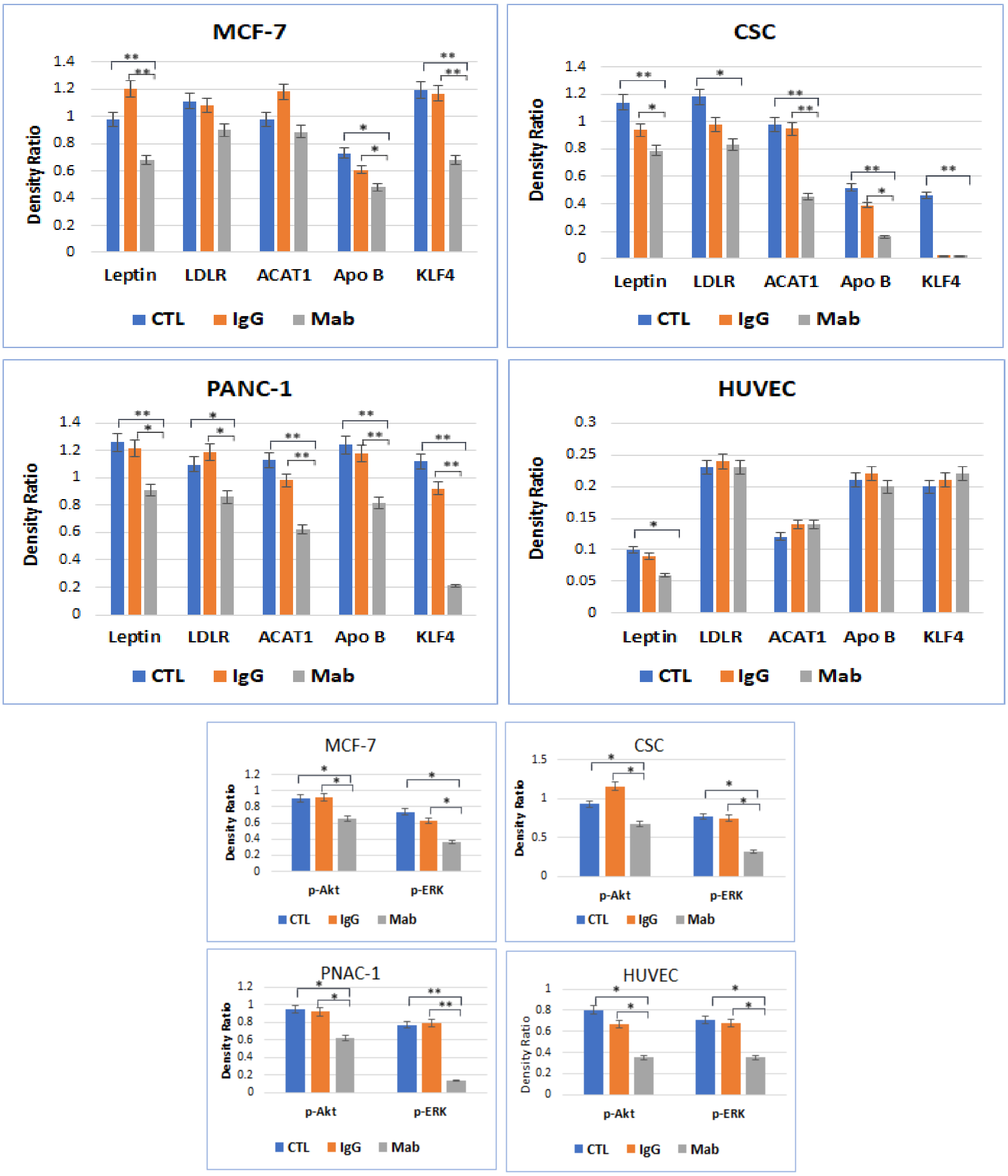

For figure 3B. Western blot of TA-MUC1 effect on cholesterol metabolism within different cancer cells is liable to restraint by the anti-TA-MUC1 antibody compare with isotype IgG (IgG) treated and untreated (CTL) cells as well. The ratio of protein band density was calculated in respect of the level of GAPDH, AKT or ERK. t-test used to determine significance, * $P < 0.05$, ** $P < 0.01$ for anti-TA-MUC1 antibody treated cells respectively compared with CTL and IgG treated cells.

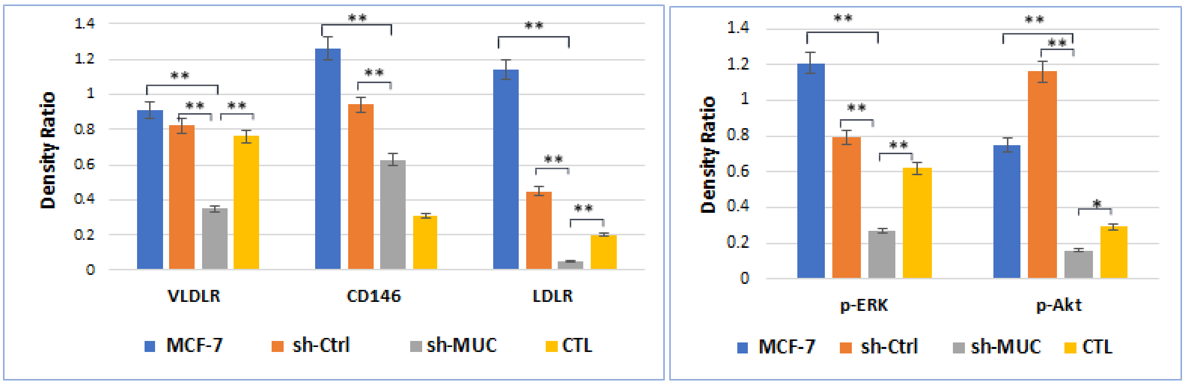

For figure 5A. Western blot of THP-1 cells respectively co-cultured with MCF-7, shCtrl-MCF7 and shMUC1-MCF7 cells for 5 days -, untreated THP-1 (CTL) as controls. The ratio of protein band density was calculated in respect of the level of GAPDH, ERK or AKT. t-test used to determine significance, * P < 0.05, ** P < 0.01 for sh-MUC1 cells respectively compared with other cells.

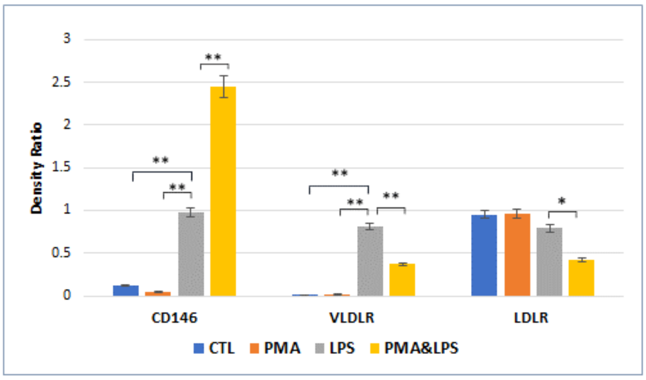

For figure 6C Western blot assay of the proteins relevant to cholesterol metabolism and foam cell formation with LPS treated macrophages (6C). The number below each Western Blot band is the level of expression calculated in respect of the level of GAPDH. t-test used to determine significance, * P < 0.05, ** P < 0.01 for LPS treated cells respectively compared with CTL, PMA or PMA&LPS treated cells.

Supplementary Figure S1

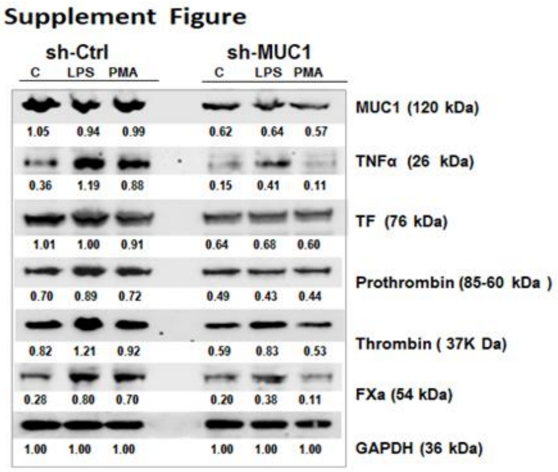

Supplementary Figure S1 Western blot assay of changes observed in a Lipopolysacharide (LPS) created inflammatory cell model. *sh-Ctrl MCF7 and shMUC1-MCF7 cells were respectively treated with LPS and PMA for 1 day. Western blot of treated cells shown that under LPS treatment, the level of inflammatory factor - tumor necrosis factor-α (TNF-α) - M1 macrophage marker was significantly increased, along with a significant enhancement in the levels of several coagulation elements, such as: TF, prothrombin, thrombin, FXa in sh-Ctrl MVF-7 cells. Such the changes were not observed in the MUC1 gene knock down cells - shMUC1-MCF7.*
